# Childhood Trauma Affects Stress-Related Interoceptive Accuracy

**DOI:** 10.3389/fpsyt.2019.00750

**Published:** 2019-10-17

**Authors:** Violetta K. Schaan, André Schulz, Julian A. Rubel, Michael Bernstein, Gregor Domes, Hartmut Schächinger, Claus Vögele

**Affiliations:** ^1^Institute for Health and Behaviour, Research Unit INSIDE, University of Luxembourg, Luxembourg, Luxembourg; ^2^Department of Psychotherapy Research, Justus-Liebig-University Gießen, Giessen, Germany; ^3^Department of Psychology and Social Sciences, Penn State Abington, Abington Township, PA, United States; ^4^Department of Biological and Clinical Psychology, University of Trier, Germany; ^5^Department of Clinical Psychophysiology, Institute of Psychobiology, University of Trier, Germany

**Keywords:** early life adversity, childhood trauma, mental health, interoception, stress, unpleasantness

## Abstract

Early life adversity (ELA) may cause permanent disturbances in brain–body signaling. These disturbances are thought to contribute to physical symptoms and emotional dysregulation in adulthood. The current study investigated the effects of childhood trauma on young adults’ interoceptive accuracy as an indicator of brain–body communication that may be dysregulated by ELA. Sixty-six participants completed an online questionnaire followed by a laboratory session including the socially evaluated cold pressor stress test during which ECG, salivary cortisol, and interoceptive accuracy were assessed. Childhood trauma was negatively related to interoceptive accuracy (IAc) after the stressor. This stress effect could not be observed for heart rate and cortisol, which were unrelated to IAc. Participants reporting higher baseline unpleasantness exhibited lower IAc after the stressor, while increases in unpleasantness due to the stressor were associated with higher IAc. Unpleasantness at baseline mediated the effect of childhood trauma on IAc after the stressor.

## Introduction

Childhood adversity can have substantial long-lasting consequences for the child concerned. Children who experienced traumatic events exhibit more mental and physical health problems in childhood and adulthood as compared to non-traumatized control participants (1–3). Existing research primarily focuses on trauma-related consequences for physical and mental health, whereas the psychophysiological mechanisms underlying these effects remain partially unclear. The current study focuses, therefore, on one candidate mechanism: the perception of bodily signals (i.e., interoception) and the potential relationship to childhood trauma.

The experience of traumatic stress during childhood can permanently alter stress responses (4–8). Chronic activation of the hypothalamic pituitary–adrenocortical (HPA) axis and the sympathetic–adreno–medullary (SAM) axis causes prolonged secretion of stress hormones that induce dysregulation of these interdependent stress axes (9), resulting in adverse effects on psychological and physical health (10). Dysregulation of the SAM axis—for example, may contribute to hypertension, whereas chronic activation of the HPA axis might result in hyper- or hyposecretion of cortisol, which is associated with major depression (11).

The activation of the physiological stress axes implies the efferent signal transmission from the brain to the body. It is likely that alterations in efferent brain–body communication also affect afferent signals on the brain–body axes and, therefore, also their perception, i.e., interoception (7). Surprisingly, until now, it is unclear if dysregulation of the physiological stress axes, as has been previously documented following early life adversity (ELA), affects interoception.

Interoception is a major determinant of mental health. The objective performance in perceiving interoceptive sensations (e.g., heartbeats) is called interoceptive accuracy (IAc) (12) and is the central facet in contemporary models of interoception (13, 14). IAc is strongly associated with the experience and regulation of emotions: individuals with higher IAc show more negative affect after a stressor as compared to those with poorer habitual awareness of their bodily state (15), suggesting stronger emotional experience with higher IAc. This observation is in line with psychophysiological emotion theories (16, 17). Intense emotional experiences increase the need for emotion regulation (18), which may be one reason why IAc is positively related to emotion regulation (19). These findings illustrate the potential of IAc as protective factor against emotional disturbance. Altered interoception is observed in mental disorders with emotional disturbances and physical symptoms, such as depression and somatoform disorders (7, 20–25). Due to the association of IAc on emotional and physical well-being, it is critical to focus on stress as accelerant and regulator of bodily and emotional states and its important effects on physical and mental ill health (26).

IAc increases after an acute stressor if attentional resources are not compromised (i.e., no competition between interoceptive and exteroceptive signals; 7, 27), but it remains unclear, which stress axis contributes to this increase. More precisely, acute stress might result in increased IAc as increases in cardiac signals through activation of SAM axis ascend to the brain and are, therefore, more easily detectable (7, 28, 29). In addition, exogenous cortisol rapidly increases heartbeat evoked potentials (HEPs), which represent an indicator of cortical representation of interoceptive signals (30). One might expect, therefore, that an increase of IAc in response to an acute stressor may be due to increase in cortisol secretion as evoked by this stressor.

In contrast to acute stress, the relationship between physiological stress axes and interoception in chronic stress remains unclear. One might speculate that chronic activation of the SAM axis might result in a state of hyper-arousal (11, 31) that can dysregulate brain–body communication (7, 32, 33) and render it more difficult for the individual to attend to specific signals of the ascending signal stream due to a lower signal-to-noise ratio. Little is known about HPA axis activation and interoception. Effects of chronic stress on the HPA axis are manifold, including diurnal hyper- or hypo-secretion, blunted reactivity to acute stressors or feedback sensitivity (34). As of today, there are only two studies addressing cortisol and interoception: one reported higher HEPs after cortisol administration (30), whereas the other found a negative relationship between baseline cortisol and HEPs (35). Although these findings suggest that cortisol affects interoceptive signal processing in the CNS, the relationship of cortisol reactivity to an acute stressor and IAc remains unclear. This is, however, of particular relevance for health and disease, as cortisol reactivity is associated with stress-related disorders (36, 37) and could affect processing of bodily sensations, as well.

Another line of argumentation associating childhood trauma and IAc has been suggested recently by Oldroyd and colleagues (38), who specifically propose that interoception is affected by social interactions. Previous studies investigating the impact of absent parental responsiveness in infancy on dissociation in young adulthood (which includes emotional and physical detachments and should therefore inhibit interoception) found that childhood verbal abuse significantly predicts dissociation later in life (39). It is plausible, therefore, to assume an association between childhood trauma and interoception.

To date, there has—to our knowledge—not been any study investigating the effects of chronic and early life traumatic stress on IAc (7). However, due to its important implications in the development of bodily related mental disorders, it is crucial to understand if and how IAc is affected by early life stress. More insight into the relationship between IAc and ELA would help in the development of prevention programs to dismantle possible stress-induced dysregulation of IAc during childhood that might increase children’s vulnerability to develop mental disorders later in life.

The aim of the current study was to investigate the impact of ELA on IAc and its relationship to acute stress reactivity. During the laboratory session, participants underwent a laboratory stress test. Autonomic stress response, salivary cortisol, and IAc were assessed at different time points before and after the stress test. We expected that (I.) IAc increases after the stressor (27). Additionally, we aimed to explore two related questions: (II.) what are the effects of ELA (i.e., childhood trauma) on IAc after an acute stressor? (III.) How is IAc related to stress-induced physiological (i.e., changes in heart rate (HR) and cortisol release) and (IV.) psychological changes (7, 15–16, 17, 28, 29).

## Method

### Participants

Sixty-six participants took part in this study. Forty-five were female, and 22 reported a parental divorce during their childhood. Mean age was 25 years (*SD* = 4.478). Most participants came originally from Luxembourg (N = 49) or Germany (*N* = 12). Two participants were born in Belgium, one in France, one in Russia, and one in South Korea. Educational background of the participants was high, with 30 having a university entrance diploma and 29 a university degree, 1 was still going to school, 1 had a certificate of secondary education, and 1 completed vocational training.

### Questionnaire Measures

Childhood trauma was assessed using the German version of the Childhood Trauma Questionnaire (40). This 28-item questionnaire (five-point Likert scale ranging from 0 = not at all to 4 = very often) consists of five subscales: emotional abuse (α = .87), physical abuse (α = .87), sexual abuse (α = .98), emotional neglect (α = .88), and physical Neglect (α = .41). The CTQ has been shown to have good psychometric properties in previous research (40). In the current study, psychometric properties were also convincing (α = .71 for the global scale). The scores indicate low to moderate childhood trauma scores in this sample, for the overall scale (*M* = 1.42, *SD* = 0.398), emotional abuse (*M* = 1.628, *SD* = 0.787), physical abuse (*M* = 1.075, *SD* = 0.263), sexual abuse (*M* = 1.088, *SD* = 0.489), emotional neglect (*M* = 2.012, *SD* = 0.819), and physical neglect (*M* = 1.319, *SD* = 0.449).

### Psychophysiological Assessment

IAc was operationalized using the Schandry heartbeat perception task (41). Participants were asked to count their heartbeats over various periods of time (25, 35, 45, 55, and 65 s) that were presented in random order. IAc was calculated using the following formula:

$$IAC=\frac{1}{5}\sum \left(1-\frac{\left(|no.of\;recorded\;heartbeats_{k}-no.of\;perceived\;heartbeats_{k}|\right)}{no.of\;recorded\;heartbeats_{k}}\right)$$

HR was used as an index of SAM axis activation and recorded continuously throughout the experiment. Electrocardiograph (ECG) signals were recorded using a precordial lead II electrocardiogram (RA, LL) with 1,000 Hz sampling rate, a hardware high-pass filter of 0.5 Hz, followed by a 0.5–35 Hz bandpass software filter. R-wave detection was automatically done in 1-min bins *via* the Mindware software’s IMP 3.1.3 module (Lafayette, OH) and controlled *via* visual inspection for artifacts by the researchers afterwards. HR was averaged during a baseline period of 5 min at the beginning of the study (baseline period), during the 3 min of the tress induction and then again 15 min after the stressor for a period of 5 min, during which participants were instructed to sit still on their chair.

HPA-axis activity was assessed in terms of salivary cortisol reactivity. Saliva samples were collected using standard absorbent swabs (Salivette, Sarstedt; Nümbrecht, Germany) by participants. Immediately following completion of the experimental session, samples were frozen at −20°C. Salivary cortisol was assessed using a time-resolved immunoassay with fluorometric detection (42).

### Acute Stress Induction

Acute stress was induced using the socially evaluated cold pressor test (SECPT) (43). An experimenter unknown to the participant entered the lab in a white lab coat and took a seat in front of the participant. He/she asked the participant to immerse their hand in a water container (water temperature 0–4°C) and to look into a video camera until requested otherwise. The participants were told that the experimenter and the camera would record their facial expression and behavior during the test. After 3 min,the test was over, and the experimenter left the room again with the video camera and the water container. Three participants of the 66 participants were excluded from analyses, as two did not keep their hand in the water for the duration of 3 min and 1 s because the water temperature was too high (i.e., 8°C).

### Psychological Stress Response

Participants were asked to rate feelings of pain, anxiety, and unpleasantness on a visual analog scale (10 cm) before and immediately after the SECPT. Participants also completed the Self-Assessment Manikins (44) for arousal and mood valence right before and after the stress test. These emotional states are assessed using cartoon images that represent varying degrees of emotion intensities. Participants rated their current emotional state by selecting the box of the cartoon image that best represented how they felt at that moment (nine-point Likert scale). Psychometric properties of this inventory are convincing (44) and ratings on this scale have been shown to correlate with psychophysiological arousal states (i.e., skin conductance, corrugator, and zygomatic activity; 45).

### Procedure

German-speaking participants were recruited online *via* social networks, through university postings and university circular e-mails. The study included a short online questionnaire and a 1-h experimental session. Participants were screened for the following exclusion criteria: cold intolerance (e.g., Raynaud’s disease), current medication, alcohol consumption >30 g/day, illicit drug intake within the last 3 months, and current mental disorders that might affect the experimental results (e.g., depression, anxiety disorder, psychosis, suicidal ideation). More specifically, participants were asked during a telephone screening, if they had any current or past mental disorder, and if they had received any treatment for this condition (if any). A female experimenter was always present during the session for safety, sitting in a cubicle outside the visual field of the participant. After signing the informed consent, participants were attached to the physiological equipment and a 5-min baseline period (t1) ensued. Then, IAc was assessed, and psychological baseline measurements (arousal, mood valence, pain, and unpleasantness) were taken. This was followed by the SECPT. Ratings of arousal, mood valence, pain, and unpleasantness were again measured immediately after the SECPT. Salivary cortisol was collected immediately before the SECPT and then at 5, 15, 25, 35, and 45 min thereafter. IAc was measured again after the second saliva collection (approx. 6 min after SECPT onset). Participants received a financial compensation of 20 euros. The study design was approved by the Ethics Review Panel of the University of [Luxembourg].

### Statistical Analysis

All data were scored and analyzed using *AcqKnowledge 4.2*, *Mindware 3.1.3*, and *SPSS* *21*. Outlier identification was carried out by visual inspection for all variables, and extreme values (> 2.5 *SD*s above the mean) were set to missing. All missing data were independent of experimental condition and considered to be missing at random. Significance level was set at *p* < .05. Effect sizes are reported for any significant interaction or main effect using *Cohen’s d* statistic (for *t*-tests) or partial eta-squared statistics (η_p_^2^; for ANOVA results). In the case of significant Levene-test results, *t*- and *F*-values for unequal variances are reported.

Firstly, three paired t-tests were calculated to investigate stress-induced changes of pain, anxiety, and unpleasantness ratings, respectively. Secondly, cortisol stress responses were analyzed by computing a repeated measures ANOVA using testing time (before the SECPT and then 5, 15, 25, 35, and 45 min after the SECPT) as within subject variable. Third, a repeated measures ANOVA was conducted to investigate changes in HR in response to the SECPT, using testing time [baseline (t1), during the SECPT (t2), after the SECPT (t3), and at the end of the experiment (t4)] as within-subject variable.

Hypothesis (I.) was analyzed using a paired sample t-test comparing IAc before and after the SECPT.

To test hypothesis (II.), the data was restructured in long format (i.e., one time point per row) allowing for binary coded time [before (= 0) and after the SECPT (= 1)], childhood trauma, the interaction of time and trauma, and baseline IAc to be entered as predictors of IAc at the respective time points (before and after the stressor). Given the coding of time, the intercept represents the average IAc baseline score across participants. Because of the inclusion of baseline IAc, changes from pre- to post-SECPT (as indicated by the coefficient of time) are adjusted for differences in baseline IAc.^1^

Furthermore, to investigate if possible stress-induced effects could also be observed for changes in cortisol or heart rate, two exploratory regression analyses were calculated, respectively. Again, time was entered into the model, as was childhood trauma, the interaction of time and trauma, as well as baseline cortisol (or heart rate) as predictors for cortisol (or heart rate). All continuous variables were z-transformed before inclusion.

To investigate if IAc is affected by salivary cortisol (HPA axis) or HR (SAM axis) before, during, and after the acute stressor (III.), Pearson correlations between IAc and salivary cortisol and HR for all time points were calculated. We also analyzed if emotional reactivity was related to IAc (IV.) by calculating correlations between IAc and change scores in unpleasantness before and after the SECPT.

To examine the associations between childhood trauma, sex, interoception, unpleasantness, cortisol, and heart rate at baseline with IAc after the SECPT, we calculated a mediation model based on a bootstrapping procedure (46). Unpleasantness, cortisol, heart rate, and IAc at baseline were entered as mediators between childhood trauma and sex (i.e., male, female) and IAc after the SECPT with n = 10,000 resamples. Bias-corrected and accelerated 95% bootstrap confidence intervals (CI) for indirect effects were calculated.

## Results

### Psychological Stress Response

Participants reported an increase in pain perception [*M*_1_ = 0.469, *SD*_1_ = 0.858, *M*_2_ = 5.231, *SD*_2_ = 2.584, *t*(62) = −14.890, *p <* .001, CI (−5.401, −4.123)], anxiety [*M*_1_ = 0.259, *SD*_1_ = 0.496, *M*_2_ = 0.549, *SD*_2_ = 1.095, *t*(62) = −2.640, *p* = .008, CI (−0.510, −0.071)], and unpleasantness [*M*_1_ = _._754, *SD*_1_ = 1.118, *M*_2_ = 4.335, *SD*_2_ = 2.708, *t*(62) = −9.610, *p* < .001, CI [−4.326, −2.836)] after the SECPT as compared to baseline. Furthermore, stress-related increases in arousal [*M*_1_ = 4.698, *SD*_1_ = 0.858, *M*_2_ = 5.302, *SD*_2_ = 1.931, *t*(62) = −3.677, *p* < .001, CI (−.931, −.275)] and a decrease in positive valence [*M*_1_ = 7.127, *SD*_1_ = 1.251, *M*_2_ = 6.539, *SD*_2_ = 1.389, *t*(63) = 3.801, *p* < .001, CI (−.278,.896)] could be observed.

### Stress Response

**Figure 1:**
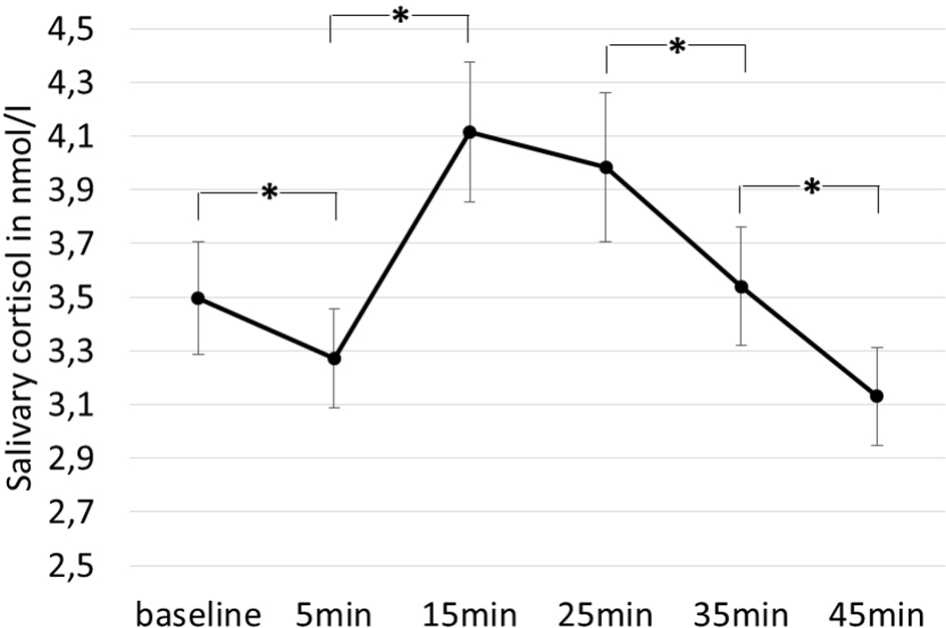
Changes in cortisol over time (baseline = t1, 5 min = t2, 15 min = t3, 25 min = t4, 35 min = t5, 45 min = t6). Error bars indicate one standard error. Stars indicate significant differences between time points.

The main effect for testing time was significant [*F*(1.534, 88.99) = 9.058, *p* = .001, η_p_^2^ = .135; see **Figure 1**] suggesting a significant change of cortisol release over time. While *post hoc* tests showed a significant decrease of salivary cortisol from t1 to t2 [*t*(59) = 3.339, *p* = .004, *d* = .10, CI(.089;.367)], there was a significant increase from t2 to t3 [*t*(59) = −2.591, *p* = .004, *d* = .349, CI(−1.29; −.401)]. Cortisol levels were stable between t3 and t4 [*t*(59) = 1.503,* p* = .552, *d* = .013, CI(−.076;.343)]. Cortisol dropped significantly from t4 to t5 [*t*(58) = 4.123, *p* < .001, *d* = .149, CI(.224;.661)] and again from t5 to t6 [*t*(59) = 4.516, *p* < .001, d = .176, CI(.306;.815)].

**Figure 2:**
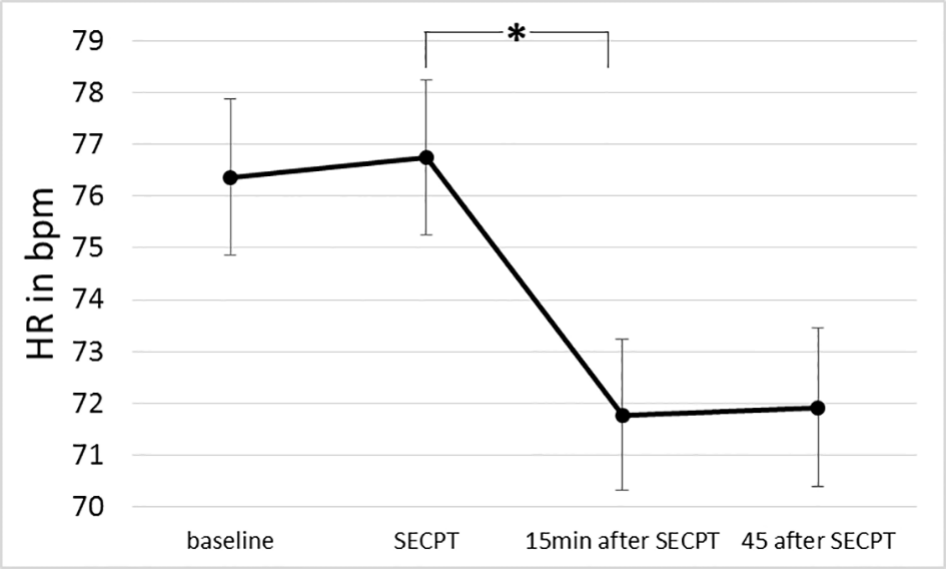
Changes in HR over time (before, during, and after the stress). Error bars indicate one standard error. SECPT, socially evaluative cold pressor test. *indicate significant differences between time points.

The repeated-measures ANOVA of HR revealed a significant main effect for time [*F*(1.819, 107.338) = 35.652, *p* < .001, η_p_^2^ = .377; see **Figure 2**]. While HR did not differ between t1 (*M* = 76.37, *SD* = 11.66) and t2 [*M* = 76.74, *SE* = 11.555, *t*(60) = .484, *p* = .630, CI(−1.963; 1.198], there was a significant decrease in HR from t2 to t3 [*M* = 71.78, *SD* = 11.329, *t*(60) = 6.522, *p* < .001, CI(3.476; 6.522)]. HR did not change between t3 and t4 [*M* = 71.92, *SD* = 11.873, *t*(59) = 0.469, *p* = .641, CI(−.768;.476)].

### Interoceptive Accuracy

IAc was higher after the SECPT (M = .768, SD = .20) compared to baseline levels [M = .721, SD = .20, t(62) = 3.039, p = .003, d = .235, CI(−.077; −.016)].

**Figure 3:**
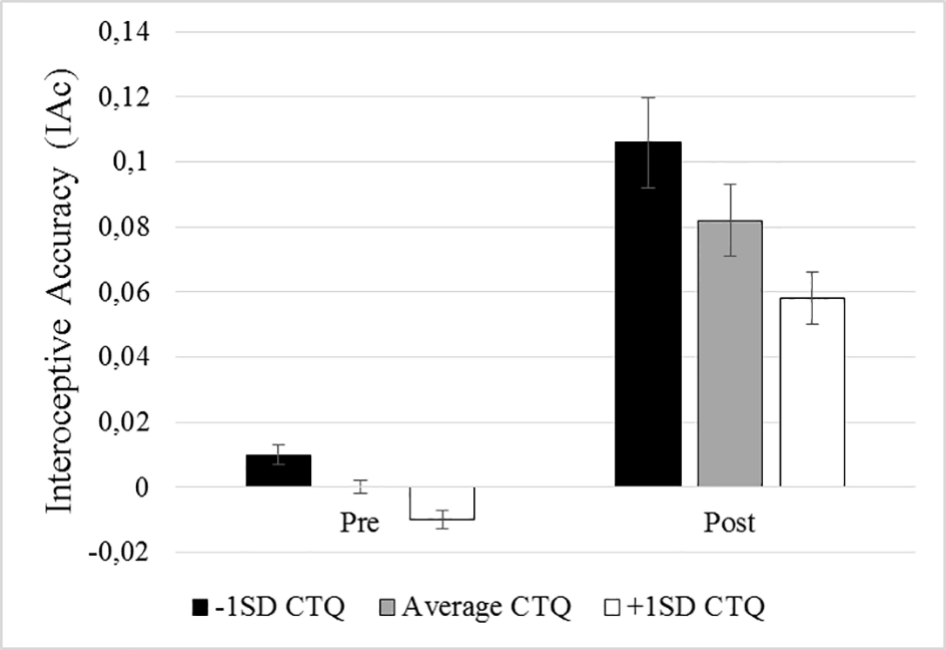
Illustration of the interaction between time and trauma. The black column represents participants scoring 1 standard deviation above the mean scores of the childhood trauma questionnaire (CTQ), the gray column represents participants with mean CTQ scores, and the white column represents participants scoring 1 standard deviation below the mean of the CTQ. Time (pre = baseline; post = after the stressor) is illustrated on the x-axis, IAc on the y-axis.

The regression analysis showed that changes from pre- to post-SECPT remained significant (β = .410, t = 2.958, p = .004) after adjustment for baseline differences in IAc and childhood trauma. Furthermore, IAc at baseline was a significant predictor for mean IAc (β = .889, t = 24.163, p < .001). The main effect for childhood trauma was not significant (β = −.005, t = −.096, p = .923), indicating no association between childhood trauma and IAc at baseline. There was a significant association, however, with time (β = −.305, t = −2.128, p = .035), indicating that higher levels of childhood trauma reduced the increase in IAc from pre- to post-SECPT (see **Figure 3**). **Figure 3** visualizes the association between time and IAc for different levels of childhood trauma (1 SD below the mean, mean, and 1 SD above the mean). An estimation of the region of significance by using the Johnson–Neyman technique (e.g., 47) indicated that, for participants scoring 1.263 standard deviations above the mean, no significant increase could be observed anymore from pre- to post-SECPT. These results even reveal a reverse relationship for childhood trauma scores 1.411 standard deviations above the mean, with post-SECPT scores being lower than pre SECPT scores.

We further analyzed if the effects observed for interoception were also reflected in physiological and hormonal stress-induced changes. We, therefore, replicated the same analyses using heart rate and cortisol instead of IAc as dependent variables in the regression analysis.

We first included HR at baseline and HR during the stress induction in the model. HR was significantly higher during the SECPT than before (β = .245, t = 2.749, p = .048). The main effect of HR at baseline was significant (β = .938, t = 28.070, p < .001). Childhood trauma did not predict mean HR (β = .013, t = 1.329, p = .780), and the interaction between childhood trauma and time was only marginally significant (β = −.239, t = −1.889, p = .061).

Second, we included HR at baseline and HR 15 min after stress induction in the model. The main effect of HR at baseline was significant (β = .961, t = 50.958, p < .001). Heart rate was not significantly different 15 min after the SECPT compared to baseline (β = −.108, t = −1.563, p = .121), after adjusting for baseline HR levels and childhood trauma. Childhood trauma did not predict HR (β = .007, t = .259, p = .796), and also the interaction between childhood trauma and time was not significant (β = −.097, t = −1.356, p = .178).

We then entered cortisol at baseline (as predictor) and cortisol 5 min after the stress induction (as dependent variable) into the model. Cortisol was significantly lower directly after the SECPT than before (β = −.156, t = −2.120, p = .036). The main effect of cortisol at baseline was significant (β = .973, t = 48.679, p < .001). Childhood trauma did not predict cortisol levels (β = −.013, t = −0.446, p = .656), and also the interaction between childhood trauma and time was not significant (β = .080, t = 1.052, p = .295).

Finally, we entered cortisol at baseline and cortisol 15 min after stress induction into the model. Cortisol levels 15 min after the SECPT were not significantly different from cortisol levels at baseline (β = .077, t = 0.400, p = .690), after adjustment for cortisol baseline levels and childhood trauma. The main effect of cortisol at baseline was significant (β = .808, t = 15.526, p < .001). Childhood trauma did not predict cortisol (β = −.017, t = −0.226, p = .822), and also the interaction between childhood trauma and time was not significant (β = .036, t = 0.180, p = .858).

### Interoceptive Accuracy and Stress-Induced Physiological and Psychological Changes

With regard to possible trauma-induced stress axis dysregulation, we calculated Pearson’s correlations between salivary cortisol and HR before, during, and after the SECPT. None of these correlations was statistically significant (*p* > .50). Furthermore, we wanted to examine if stress-induced changes in unpleasantness during the SECPT were related to IAc after the stressor. Indeed, unpleasantness (IAc baseline: *r* = −.294, *p* = .017; IAc after the SECPT: *r* = −.421, *p* < .001) as well as stress-induced changes in unpleasantness (i.e., psychological reactivity; IAc after the SECPT: *r* = .277, *p* = .025) were related to IAc.

**Figure 4:**
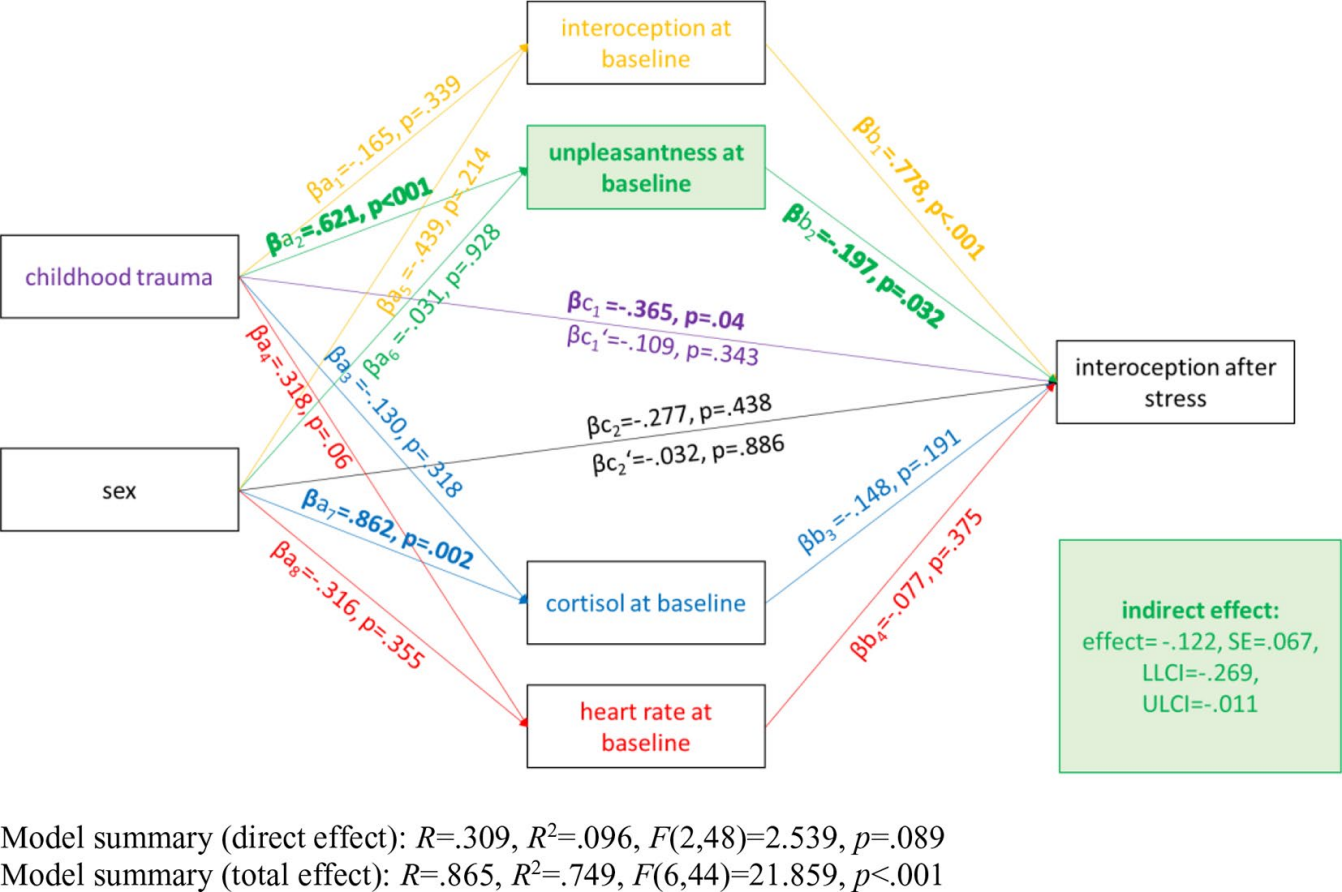
Illustration of the bootstrapping analysis. Paths are represented using the following standardized coefficients: a_1_ to a_8_ indicate the coefficients from childhood trauma and sex to each mediator (i.e. interoception at baseline, unpleasantness at baseline, cortisol at baseline, and heart rate at baseline) respectively; b_1_ to b_8_ are the respective coefficients from the mediators (i.e., interoception at baseline, unpleasantness at baseline, cortisol at baseline, and heart rate at baseline) to interoception after the stressor; c is the coefficient that indicates the total effects of childhood trauma/sex on mental health symptoms without controlling for the mediators and c’ reflects the path from childhood trauma/sex to interoception after the stressor controlling for the mediators (direct effects).

To further examine the mechanisms underlying these findings, we calculated a mediation model (see **Figure 4**). The results show that unpleasantness at baseline mediated the effect of childhood trauma on interoception after the stressor. Changes in unpleasantness, and unpleasantness after the stressor, no longer predicted interoception after the stressor, when entered into the model. Neither did cortisol nor heart rate after the stressor predict interoception after the SECPT. The final model therefore only included baseline measurements.

## Discussion

The current study is the first to investigate the effects of traumatic childhood experiences on IAc before and after an acute stressor. This is of particular relevance, as early life stressors may affect brain–body communication, which is assumed to contribute to mental disorders associated with physical symptoms (e.g., 7, 20, 24, 25). After the SECPT, participants reported increases in pain, unpleasantness, and anxiety. Furthermore, cortisol significantly increased 15 min after the SECPT. HR dropped significantly 15 min after the stressor.

This study provides further evidence for the negative impact of childhood trauma on an individual’s well-being in adulthood and suggests some potential mechanisms with regard to interoception. Childhood trauma significantly influenced IAc after the stressor: the more childhood trauma participants reported, the more difficult it was for them to perceive their heartbeat after the stressor. Interestingly, this stress effect could not be observed for our physiological measures (HR, cortisol) that were also not associated with IAc. One explanation for this pattern might be, therefore, that changes in IAc after an acute stressor are mainly a result of stress coping than of physiological changes. Interoception is theorized to be linked to the perception of emotional states and emotion regulation (15, 17, 19, 48) and—in this study—was related to unpleasantness rating. Participants reporting higher baseline unpleasantness showed lower IAc, while increases in unpleasantness due to the stressor lead to higher IAc. The latter finding is in line with study results from Kindermann and Werner (15), who found that individuals with higher IAc experienced more negative affect after an acute stressor. These opposing effects suggest trait and state differences in unpleasantness. Increases in unpleasantness might reflect stress-induced attentional shifts toward internal changes and therefore better sensitivity to physiological changes (e.g., increase in HR) facilitating perception of heartbeat (28, 49). The increased internal focus during stress may reflect an adaptive stress response, as it allows the assessment of bodily changes and stress-induced emotional states (17). This is supported by findings that the ability to correctly identify emotions is associated with higher IAc (50). The negative association between baseline unpleasantness and interoception is in line with studies observing a negative relationship between depression and IAc (51), as depressed individuals experience events as more unpleasant and report more negative and less positive affect during daily life (52). Baseline unpleasantness might represent a trait personality factor. Trait unpleasantness might, therefore, represent a chronic emotional state that is no longer informed by bodily signals. The mediation model indicates that the relationship between childhood trauma and interoception might be fully mediated by unpleasantness at baseline with participants who experienced more childhood trauma reporting more trait unpleasantness and trait unpleasantness being negatively related to interoception after the stressor.

As the childhood trauma scores in the current sample were low to moderate, future studies should focus on severely traumatized populations to clarify if ELA may affect brain–body communication in these conditions. We could replicate previous findings in that IAc as measured with the Schandry paradigm increases after an acute stressor (27). Importantly, IAc after an acute stressor was negatively associated with childhood trauma, with participants scoring higher on the CTQ showing lower IAc than those with lower CTQ scores. This supports current models of chronic stress induced malfunctions of neural circuits underlining successful body–brain communication (7, 32, 33).

These results could, therefore, reflect the impact of high-intensity chronic stress on long-term changes in stress system functioning and trait emotionality (53). High-intensity, threatening, and chronic stresses may result in overwhelming physiological and cognitive reactions (53–56) that might not allow the individual to identify specific bodily signals. In line with this interpretation, reduced HEPs have been observed in individuals with borderline personality disorder (57), a disorder known to be highly associated with the experiences of traumatic events (58–60). Threatening situations overcharge an individual’s coping abilities and consequently result in freezing and numbing (54, 55). Threat reactions might therefore result in suppression and denial of emotions and bodily symptoms as survival coping mechanism (54) and might explain why childhood trauma is associated with dissociation (that includes emotional and physical detachments and should therefore inhibit interoception) in later life (39) and, in this study, with reduced IAc after an acute stressor.

This is in line with Oldroyd and colleagues (38) who recently argued that social interactions can importantly affect interoception: while caregivers can—following their model—help the development of accurate interoception by validating their child’s bodily experiences, they can also motivate children, through neglect and abuse, to avoid their bodily feelings. Neuroimaging studies provide evidence of shared neuronal regions of early attachment related experiences and interoception, such as the anterior cingulate cortex (61, 62) or the orbitofrontal cortex (63–67). While the orbitofrontal cortex, for instance, has not only been associated with early caregiving experiences and attachment (68), it has also been found to be implicated in the interpretation of bodily signals and affect regulation (68, 69). Indeed, attachment stiles are associated with interoception, as avoidant individuals reported lower interoceptive functioning (38). Furthermore, parental rejection of negative emotions was negatively related to the congruency of self-reported negative emotions and physiological distress signs (38). Since traumatic childhood experiences, such as emotional and physical abuse or neglect, are related to insecure attachment stiles that have protective value within the context of their families (70), one might expect an association between childhood trauma and interoception as well. This is corroborated by the current results, which lend further support to recent research relating early family experiences with interoceptive functioning (38, 39), while providing new knowledge on the association between childhood trauma and IAc after an acute stressor.

The finding of a negative association between childhood trauma and IAc after an acute stressor and its mediation through unpleasantness at baseline provides a theoretical foundation for prevention programs. It is important to help children to emotionally adapt to their traumatic experiences. Long-term changes in affect are often encountered after chronic stress and are even a diagnostic criterion for diagnoses such as post-traumatic stress disorder or personality change after catastrophic experience (71). It seems crucial to buffer the effect of childhood trauma on negative emotionality, by helping those experiencing childhood trauma to correctly integrate the traumatic experience into their biographic memory—for example, by using reframing techniques to reduce feelings of guilt, shame, and disgust. Additionally, techniques of enhancing non-evaluative mindful attention might help to enable children to better perceive their internal states. It seems important to help children who experienced childhood trauma to increase awareness to their stress-induced bodily and emotional changes over time and thereby enabling them to better differentiate cardiac signals from the stream of ascending bodily signals. This may have helped them to (a) better differentiate emotions from bodily changes and thereby to (b) better perceive and regulate their emotions.

## Limitations

Limitations of the present study concern its cross-sectional design and the preponderance of students and female participants, which restricts the generalizability of the results to the general population. Questionnaire data such as childhood trauma was assessed retrospectively and might therefore be susceptible to memory bias. Future studies could use longitudinal designs to better understand the developmental trajectories leading to increased vulnerabilities for mental or physical disorders. Recently, potential shortcomings of the heart beat counting task have been discussed, such as low correlations between actual and perceived heart rates, or the influence of personal beliefs about one’s heart rate and the ability of time perception on IAc (72, 73). Despite these potential shortcomings, the Schandry heartbeat perception task is a widely used, well-established measure in this field, whose validity was supported by a substantial overlap with neurophysiological indicators of interoceptive signal processing (i.e., heartbeat-evoked potentials) (74–77), as well as by a reduction in individuals with a degeneration of afferent autonomic nerves (78). Furthermore, it has been shown valuable in the identification of abnormal interoception in different mental disorders (20, 23). 

## Conclusion

This study reveals that childhood trauma is associated with lower IAc after an acute stressor, which may be explained by higher trait unpleasantness. The findings support current models of chronic stress induced malfunctions of neural circuits underlying successful brain–body communication. This finding may facilitate the development of prevention strategies targeting children who experienced childhood trauma with the aim to raise awareness to stress-induced bodily changes over time and thereby enabling them to better differentiate cardiac signals from the stream of ascending bodily signals. This may have helped them to (a) better differentiate emotions from bodily changes and thereby to (b) better perceive and regulate their emotions.

## Data Availability Statement

The datasets generated for this study are available on request to the corresponding author.

## Ethics Statement

The study was reviewed and approved by the Ethics Review Panel of the University of Luxembourg. The participants provided their written informed consent to participate in this study. 

## Author Contributions

Conceptualization: VS, AS, MB, HS, CV. Formal Analysis: VS, JR. Funding Acquisition: VS, AS, GD, CV. Investigation: VS. Project Administration: VS, AS, CV. Visualization: VS, JR. Validation: AS, CV. Writing – Original Draft Preparation: VS. Supervision: AS, MB, GD, HS, CV. Writing – Review and Editing: VS, AS, JR, MB, CV. Methodology: VS, AS, MB, GD, CV. Resources: GD, CV.

## Funding

The University of Luxembourg, the University of Trier and the Fonds National de la Recherche Luxembourg (FNR) funded this research (AFR PhD fellowship No 9825384). The funding bodies were neither involved in the study design, nor in the collection, analysis, or interpretation of the data.

## Conflict of Interest

The authors declare that the research was conducted in the absence of any commercial or financial relationships that could be construed as a potential conflict of interest.

## Footnotes

1 The law of initial values predicts a systematic effect of baseline values on reactivity scores in that lower baseline scores are associated with larger increases to stress (i.e., negative correlation). It is, therefore, important to adjust change scores for baseline levels.
